# International collaborative actions and transparency to understand, diagnose, and develop therapies for rare diseases

**DOI:** 10.15252/emmm.201910486

**Published:** 2019-04-12

**Authors:** Kym M Boycott, Lilian PL Lau, Christine M Cutillo, Christopher P Austin

**Affiliations:** ^1^ Children's Hospital Eastern Ontario Research Institute University of Ottawa Ottawa ON Canada; ^2^ IRDiRC Scientific Secretariat Paris France; ^3^ National Center for Advancing Translational Sciences (NCATS) National Institutes of Health (NIH) Bethesda MD USA

**Keywords:** Biomarkers & Diagnostic Imaging, Genetics, Gene Therapy & Genetic Disease

## Abstract

Rare diseases, which affect over 350 million people worldwide and frequently go undiagnosed or misdiagnosed for years, suffer from sparse and dispersed medical knowledge leading to even rarer approved and effective therapeutic options for patients. A vast, unmet need for research and investment to advance diagnostic capabilities and therapeutic development must be confronted, despite the myriad of challenges faced. Several fundamental shifts are changing the landscape of rare diseases research and development, particularly with the application and extension of results to common diseases and the advancement of personalized medicine initiatives. Collaborative strategies that pool resources and knowledge are vital, including team science, research networks, novel funding models, shared knowledge platforms, and innovative regulatory frameworks. Importantly, patients are also increasingly involved as research partners and funders, pushing for open science and transparency, and breaking down data silos and geographical borders, often enabled by online platforms accessible from across the globe. The International Rare Diseases Research Consortium (IRDiRC), established in 2011, has been working diligently to unify stakeholders (e.g., funding bodies, companies, umbrella patient advocacy groups, researchers, and experts) to seek and drive solutions that aim to accelerate diagnosis and therapeutic development for rare diseases worldwide. Further and future advances will depend on continued collaborations and cooperation among stakeholders, working hand in hand with patients, and exponentially improving research and development efficiency. Critically, engagement with stakeholders from underrepresented populations and less‐developed countries must be prioritized, to enable all people living with a rare disease to receive an accurate diagnosis, care, and therapy.

Two sisters were born 30 years ago and developing normally until their second birthday when they began to suffer from relentless seizures. They started to lose their motor skills, became unsteady on their feet, and their ability to communicate faltered. Despite extensive investigations throughout their childhood, no diagnosis of their condition could be made. At the age of 25 years, they suffered from seizures on a daily basis and both needed wheelchairs. As new and powerful sequencing technologies became available, their doctor decided to sequence the coding regions of their genomes and the young women were eventually diagnosed with cerebral folate transport deficiency, a rare recessive condition that was only identified in 2009 and reported in < 25 people in the world since. They were started on a high‐dose folinic acid therapy, and their seizures decreased by half; the prior anti‐epileptic medications could be reduced (Ferreira *et al*, 2016). The long diagnostic odyssey of these sisters is by no means uncommon, and earlier diagnosis and treatment would have had a significant impact on their neurodevelopmental outcome.

While there is no single global definition, a condition affecting fewer than 1 in 2,000 to 10,000 patients is generally considered a rare disease. Although rare diseases are individually uncommon, they affect, in aggregate, more than 350 million people worldwide, approximately half of whom are children. There are an estimated 6,000–8,000 rare diseases of which about 80% are likely genetic in origin. Symptoms and severity differ, but most patients share the fate of the two sisters. Rare diseases are frequently undiagnosed or misdiagnosed for years, the knowledge of many conditions is sparse at best, and effective therapies are even more rare. Less than 6% of rare diseases have an approved treatment option reflecting, in part, the long‐tail distribution of their prevalence and the understandable reluctance of biotech and pharmaceutical companies to invest into diseases for which little is known in terms of causes and mechanisms. Compounding the issue, those companies that do invest compete for a handful of well‐known, well‐studied, and relatively prevalent diseases to ensure a return on their investment. This leaves a vast, unmet need for research and investment to further understand the underlying mechanism of most rare diseases, to advance diagnostic capability, and to decrease the risk of therapeutic development.

The challenges for research are myriad. The patient populations are typically small, heterogeneous, and geographically scattered, and the natural history and biological understanding of their disorders are limited. There are few and unevenly dispersed medical experts and a lack of well‐defined clinical care standards. Additionally, even if patients would like to be included in research, it is difficult to connect them to biomedical sample collections and registries, which perpetuates a scarcity of patient samples and data. More tools, including cell, tissue, and animal models, are needed to better understand disease mechanisms and progression. The recruitment and retainment of patients for clinical trials pose an additional hurdle. Researchers also face obstacles to obtain funding for activities such as natural history data collection, maintenance of tools and infrastructures, and running trials.

Fortunately, this dire situation is beginning to change, owing to at least three fundamental shifts. First, rather than being studied as isolated disorders, rare diseases are increasingly characterized by commonalities, and diagnostic and therapeutic platforms are being developed that would be applicable to many or all rare diseases. Second, patients are increasingly becoming involved as research partners and funders, and bring urgency, relevance, and focus to research efforts to develop better diagnostics and treatments. Third, cooperation and coordination among funding agencies, scientists, companies, and patient groups worldwide are beginning to overcome some of the challenges of dispersed patients and researchers, and limited funding. Moreover, the lessons learned from research on rare diseases are applicable to many common diseases and valuable for implementing personalized medicine initiatives.

## Strategies to understand, diagnose, and develop treatments

### Team science and research networks

The dispersion of stakeholders and the wide diversity of research disciplines required for an effective understanding, diagnosis, and therapy development of any rare disease, much less all of them, dictate the need for a team strategy on a national level and an international level. Individual investigators make valuable, but limited contributions, whereas a team collaborates instead of competing for data from a scarce patient population. It is critical that results and data are published and made publicly available for the benefit of the field—and thereby patients—as a whole. This includes responsible data sharing that adheres to the FAIR (Findable, Accessible, Interoperable, and Reusable) principles (Wilkinson *et al*, 2016); indeed, research networks and initiatives increasingly adopt the principles of open science with publicly accessible resources, tools and knowledge bases, and enhanced sharing of medical research data with appropriate oversights.

In Canada, for example, the Care4Rare initiative connects 21 academic sites to a super‐team of collaborating clinicians, researchers, scientists, and bioinformaticians, who work on improving the diagnosis and treatment of rare diseases for patients not only in Canada, but around the world. In 6 years, they have studied more than 1,000 rare diseases, enabled a diagnosis for over 1,000 families, and discovered nearly 130 novel disease‐causing genes.

In Europe, a collaborative research network of about 100 clinicians and researchers from ten countries form the EURenOmics consortium to advance research on rare renal diseases. In 5 years, they have collectively discovered 26 new disease genes and 11 new genomic rearrangements; developed new diagnostic tests, four of which will be made commercially available; identified a new treatment for 10% of patients with genetic nephrotic syndrome; and built a biobank with samples from more than 20,000 patients. In the same 5‐year period, the NeurOmics consortium, that involves 24 academic and industry partners in Europe and abroad, discovered over 100 new disease genes, developed eight diagnostic panels covering over 1,600 genes, and sponsored five therapeutic trials. Additionally, RD‐Connect—a unique infrastructure to facilitate rare disease research by allowing researchers and clinicians to share genomic, phenotypic, registry, and biobanking information—includes data generated by both the EURenOmics and NeurOmics consortia, and many other research partners.

In the USA, the Centers for Mendelian Genomics have collaborated with more than 2,000 investigators in 82 countries to collect almost 60,000 samples and identify over 1,200 new disease genes. The Rare Diseases Clinical Research Network (RDCRN), first established in 2003, brings together over 2,500 researchers from many disciplines, 22 clinical research consortia that each studies a group of related rare diseases, a common data management center, and more than 130 patient advocacy groups to investigate more than 200 rare diseases. The success of each of these initiatives and their advances in the field resulted from strong collaboration and sharing among partners that produced scientific insights and patient‐relevant advances that would have been impossible for any one partner to achieve alone.

### Collaborative and novel funding models

Public funders are also increasingly collaborating. For example, the E‐Rare 3 initiative, co‐funded by the EC and 26 partners in 18 countries from Europe, Canada, Israel, and Japan, finances transnational collaborations to advance progress of rare disease research from bench to bedside, to enable knowledge transfer across borders, and to create vital infrastructures including biobanks and registries (Julkowska *et al*, 2017). Yet, as the majority of rare diseases still lack research programs to understand their underlying biology, additional sources of funding are needed. Patient organizations such as the National Organization for Rare Disorders (NORD) in the USA and Fondazione Telethon in Italy raise funds and provide grants for research programs. Some have reinvented themselves as non‐profit enterprises. For example, AFM‐Téléthon created YposKesi, a pharmaceutical manufacturer of gene and cell therapy drugs for rare diseases. Other novel funding models are also being introduced to complement public and private support, often driven by patients and their advocates. Crowdfunding of specific projects, often for early proof‐of‐concept research, is another avenue explored by investigators. RE(ACT), the Rare Genomics Institute, and Find‐A‐Cure are among those that seek crowdfunding to support research. The funds raised and funneled into research programs from such efforts often help to de‐risk subsequent drug development, thereby broadening the disease areas that biopharmaceutical companies are willing to work on.

### Shared knowledge platforms

To further advance information and project sharing, multi‐institute, multi‐country and multi‐stakeholder initiatives, and infrastructures have been developed. By way of example, Orphanet, which was established in 1997 in France and is now a consortium covering more than 40 countries, gather information and maintain a central knowledge database of rare diseases and their classifications, an inventory of orphan medicines, as well as directories of expert centers and patient organizations, research projects, clinical trials, registries, biobanks and diagnostic laboratories. Orphanet recently published a report of key resources pertaining to rare diseases research in Europe, aiming to encourage adoption and, thus, maximize the return on investment and the sustainability of such platforms.

### Innovative regulatory frameworks

Innovative regulatory frameworks for rare disease therapeutic development have played a crucial role in increasing the pace of drug approvals in many countries. The enactment of the Orphan Drug Act in the USA in 1983 as well as the EC Regulation 141/2000 in 2000 has attracted biopharmaceutical investments through regulatory and economic incentives, including market exclusivity, fee waivers, accelerated review, protocol assistance, and scientific advice. Open discussions between regulators, sponsors, researchers, and patients about clinical trial designs with small patient populations are enabling synergistic collaborations to increase the cost‐effectiveness of studies and reduce their development time. Additionally, governments and regulatory bodies in many countries have begun to encourage or require the input of patients in this process. Their perspective enhances the understanding of key aspects of their disorders, outcome measures relevant to them, and risk–benefit analysis of potential interventions. Real‐world evidence collection also depends on patient involvement and retention. Regulators and health technology assessors are increasingly stressing that sponsors of clinical studies should include patient‐centric methods in their protocol designs.

### Patients as partners

Rare disease patient advocacy organizations are leaders in the patient‐centric trends that are also seen for more common diseases. Rare disease patients and their caregivers have been enormously enabled by the Internet, as it has helped them to connect across the globe and share care and research strategies. They are breaking down data silos as well as geographical borders, and in the case of RareConnect, language barriers, opening up the possibility of direct contact with investigators for research and clinical studies. A multitude of applications are also available for smartphones to track health conditions and collect epidemiological data, which are extremely valuable for natural history studies—a vital element of drug development and approval. Patients are becoming true partners in the care and research for their diseases; such “activated patients” typically have better disease management and better clinical outcomes (Greene *et al*, 2015). Their engagement is open science at its core, and it is critical for research.

## IRDiRC and its collaborative efforts

The International Rare Diseases Research Consortium (IRDiRC) was established in 2011 based on a joint commitment to rare disease research by the EC and the US NIH. IRDiRC operates based on principles of transparency and has been coordinating resources, promoting best practices and policies, and, importantly, driving solutions to accelerate diagnosis and therapeutic development worldwide (Dawkins *et al*, 2018). This unifying passion and focus has brought together nearly 60 national and international governmental and non‐profit funding bodies, companies, and umbrella patient advocacy groups as members, along with hundreds of collaborating researchers and experts from all over the globe.

**Figure 1:**
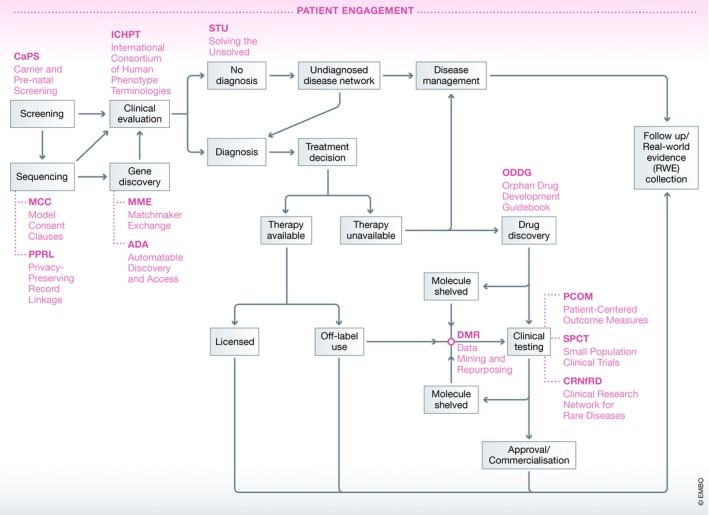
**The rare disease research roadmap and actions by** **IRD** **i** **RC** **to facilitate diagnoses and therapies for rare diseases**.

The average diagnostic odyssey faced by a patient is about 7 years, and only a small fraction of rare diseases have any therapy approved by a regulatory body. Thus, to change the narrative around rare diseases as inherently untreatable or even unimportant, IRDiRC announced a new vision in 2017 that would “Enable all people living with a rare disease to receive an accurate diagnosis, care and available therapy within 1 year of coming to medical attention”—along with three 10‐year goals for the Consortium to make substantial progress toward this vision (Austin *et al*, 2018). IRDiRC works in multiple dimensions to generate knowledge, ethically share research data, promote collaborative research infrastructures, and de‐risk investment in rare diseases research. The key driver through which IRDiRC effects change is its steadily growing number of Task Forces that work on specific, time‐limited topics, identified by members of the Consortium's Scientific and Constituent Committees (Fig 1). Often, these teams cooperate with other partners, such as the Global Alliance for Genomics and Health (GA4GH) and Orphanet.

### Actions to facilitate and improve diagnosis

The Diagnostics Scientific Committee (DSC) spearheaded a number of activities to facilitate and improve diagnosis. The Matchmaker Exchange (MME) was created to support and facilitate gene discovery through the creation of a federated network of phenotype and genotype databases. The coding and classification of diseases based on a standard nomenclature were also identified as key factors for accurate diagnoses, appropriate care, and data collection. One of the earliest initiatives in this field was the effort of the International Consortium of Human Phenotype Terminologies (ICHPT), a collaborative action led by representatives from IRDiRC, Orphanet, Online Mendelian Inheritance in Man (OMIM), and Human Phenotype Ontology (HPO). ICHPT creates a list of phenotypic terms that represent major abnormalities encountered by rare disease patients. It also cross‐references several existing terminologies, including the HPO, which has become the *de facto* standard in describing human phenotypes. ICHPT was adopted by many rare genetic disease databases such as DECIPHER and PhenomeCentral. Additionally, Orphanet has produced Orphacodes, a set of nomenclature to make rare diseases more visible within health and medical information systems. This set of nomenclature was used as a template for the 11^th^ revision of the World Health Organization's International Classification of Diseases (ICD) and has been integrated into public hospitals’ health records in France; work is underway to enable its implementation in other European countries following recommendations by the EC Expert Group on Rare Diseases.

Despite best efforts to enable diagnoses for all rare diseases, there remain a non‐trivial number of cases for which their underlying causal mechanism remains elusive. A Task Force devoted to Solving the Unsolved (STU): Beyond the Exome came together to describe the state of play of gene discovery, hypothesize mechanisms which may underlie unsolved cases intractable by exome sequencing, and prioritize research areas.

### Actions to improve international data sharing

To address issues related to data sharing and use with appropriate consent, IRDiRC—in particular its Interdisciplinary Scientific Committee (ISC)—works closely with GA4GH and Public Population Project in Genomics and Society (P^3^G) on a number of Task Forces. The Automatable Discovery and Access (ADA) Task Force created the ADA‐Matrix (ADA‐M, read as “Adam”), an information model/standard for consent and data use. ADA‐M has been integrated, in whole or in part, by a number of organizations including Australian Genomics Health Alliance and the Solve‐RD project, and is being actively evaluated by others including Genomics England and the EU's Biobanking and Biomolecular Resources Research Infrastructure (BBMRI).

Meanwhile, the Privacy‐Preserving Record Linkage (PPRL) Task Force explored a potential solution to reliably link patients’ datasets from multiple independent research projects while addressing ethical and legal concerns in relation to privacy and data protection. The European Unified Patient Identity Management (EUPID) approach, identified as a solution by the PPRL Task Force, provides a basic framework with potential use for federated datasets; its creators are current partners in understanding and adapting the framework for the mission of the PPRL Task Force. Discussions by the ADA and PPRL Task Forces pushed the need for consent clauses specific to rare disease research to the forefront. The Model Consent Clauses (MCC) Task Force was formed to act on this topic through the identification and definition of core consent elements that enable harmonized, interoperable data sharing and facilitate research from diverse clinical sites around the world. It aims to publish its guidelines in early 2019.

### Actions to improve development of therapies

IRDiRC Task Forces under the guidance of the Therapies Scientific Committee (TSC), have also addressed bottlenecks in drug development. Clinical trial outcome measures had often been selected with insufficient attention to the needs of patients; a Patient‐Centered Outcome Measures (PCOM) Task Force was set up to tease out the underlying challenges and propose recommendations to enable developments of PCOMs in rare diseases. However, outcome measures form only a part of the challenge in conducting rare disease clinical trials—the size of trials also matters. With often geographically dispersed and small patient populations, undertaking a standard, randomized controlled trial for a rare disease is not trivial. The Small Population Clinical Trials (SPCT) Task Force investigated the use of non‐conventional statistical methods on small population trials with the input of regulatory agencies. Three relevant EC‐funded projects will also contribute best practices in this area: Advances in Small Trials Design for Regulatory Innovation and Excellence (ASTERIX), Integrated Design and Analysis of clinical trials in small sample population groups (IDeAl), and Innovation in Small Population Research (InSPiRe).

Another aspect of therapy development, which has garnered much attention in recent years, is the mining of data and knowledge to identify new therapeutic targets and repurpose existing molecules. The Data Mining and Repurposing (DMR) Task Force identified a number of strategic infrastructure areas with the potential to accelerate research productivity and drug development, as well as strategies to increase data mining and repurposing opportunities. To address the specific needs of drug developers, the TSC is in the process of building a guidebook on the available tools and initiatives specific to rare disease drug development.

### Actions to facilitate stakeholder engagement and coordination

Member organizations of IRDiRC are also organized into Constituent Committees of Funders, Companies and Patient Advocacy groups, which tackle research barriers and identify needs pertinent to their constituencies. At present, relevant member organizations are actively providing data on research projects and clinical trials, which they have funded and are funding, to facilitate global analysis of research and clinical trials based on research type, medical domain, and disease categories. In parallel, the Funders Constituent Committee (FCC) is developing a tool to coordinate and prioritize funding efforts, to enable management and coordination of the distribution of resources on a global scale, advance prioritized topics, and avoid unnecessary duplication of similar research projects. The FCC will also set up a Task Force to analyze the existing ecosystem of national and supranational clinical research networks, and develop guiding principles for an international collaborative framework with respect to best practices, interoperability, tools, and common goals.

As patients are increasingly involved in and asked to participate in research and development, the Patient Advocates Constituent Committee (PACC) will develop a multinational, multi‐stakeholder scan to identify barriers to patient participation and develop guidelines on how best to empower and engage them through every step of research, from bench to bedside. The Companies Constituent Committee (CCC) has prioritized the problem of expanding rare disease therapy development beyond the set of better‐known diseases, which most companies are working on. A strategy to characterize the data package that has de‐risked previous rare diseases sufficiently to attract commercial activity, and then identify and fill out those data for additional diseases, is currently being formulated as a pre‐competitive collaborative effort.

### Actions to share resources to facilitate research

Finally, IRDiRC promotes the use of research tools and guidelines already available and adopted by the rare disease research community. To this end, IRDiRC introduced the Recognized Resources initiative, which provides a peer‐reviewed quality indicator that highlights standards, guidelines, tools, and platforms to accelerate the pace of discoveries and translation (Lochmuller *et al*, 2017). IRDiRC also collaborates with the Human Variome Project (HVP) for cross‐recognition of resources important in advancing the goals of both organizations.

### Actions to outcomes

**Figure 2:**
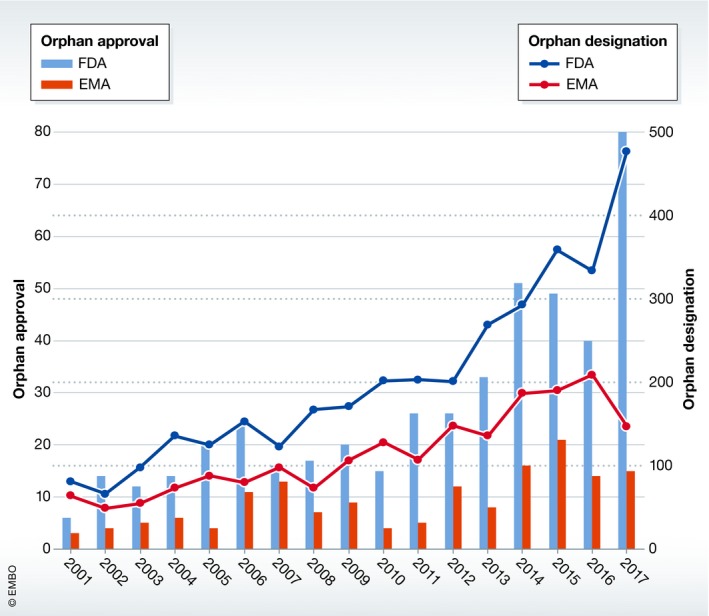
Orphan drug designations and approvals by the FDA and the EMA between 2001 and 2017 The numbers of both designations and approvals have steadily increased over the years. An orphan drug designation application may be submitted at any point of the drug development process, and when obtained, confers a number of benefits including protocol assistance, a number of financial incentives, and eligibility for marketing exclusivity—the latter comes into effect when marketing approval is awarded.

These efforts have led to further progress of initiatives: public funders have put out an increasing number of collaborative calls and research initiatives focused on rare diseases; new policies in countries previously without rare disease‐specific legislation are coming to fruition; a significant rise has been observed in orphan drug designations and approvals (EMA, 2018; FDA, 2018; Fig 2); and new and improved technologies have enhanced data sharing, resulting in faster discovery of causative genes and enabling advanced diagnostics.

## Role of stakeholders moving forward

The two sisters with cerebral folate transport deficiency benefitted significantly by appropriate diagnosis and treatment. However, this was only accomplished through scientific and medical progress achieved within their lifetime, and such progress is still needed for thousands of other rare diseases. The causative gene is still unknown for about half of all rare diseases, and while rate of therapy development is increasing, the translational rate of these developments is still low—to this day, more than 90% of rare diseases lack a specific treatment. The field needs to collectively work together to fundamentally change this calculus and accelerate progress for millions of patients waiting for science to reach them.

Future advances will depend on new tools and resources being made available to all interested stakeholders; on research funders better coordinating their programs to avoid unintended duplication and to focus on research gaps; on biopharmaceutical companies cooperating in therapy development; and on patients, regulators and health technology assessors as partners. Patients and patient advocacy organizations play a crucial role as experts, necessary partners, and collaboration brokers. International networks all have a role to play in the evolving rare disease research ecosystem. As progress accelerates, it will be critical to reach out to stakeholders from underrepresented populations and less‐developed countries, for whom the challenges of rare disease diagnosis, therapy development, and care are amplified.

To researchers, patients, companies and policy makers working in isolation, the challenges of rare disease research can be daunting. At the current rate of diagnosis and treatment development, it will be hundreds of years before prompt and accurate diagnoses and safe and effective therapies are available for all patients. Nevertheless, a common vision and collaborative actions hold enormous potential. The alignment of technologies and policies is helping to bridge geographical and data governance barriers; international scientific communities and experts are identifying the biggest roadblocks to efficient and effective research, and proposing innovative solutions to overcome them; research funders aim to close basic and translational research gaps; and patients and their families are increasingly engaged as active research collaborators for it is through their biosamples and medical records that researchers and drug developers get the keys that will unlock further progress. A dynamic global rare disease community is committed to bring these aspects together, and, as a growing community, we also welcome input on how to expand our collaborative efforts and work transparently to improve our understanding of rare diseases, and develop diagnostic tests and therapies for patients worldwide.

## Disclaimer

The views expressed in this publication are the sole responsibility of the authors and do not necessarily reflect the views of their affiliated organizations. CPA contributed in his capacity as Chair of the International Rare Diseases Research Consortium (IRDiRC), not in his role as Director of the National Center for Advancing Translational Sciences (NCATS).

## Conflict of interest

The authors declare that they have no conflict of interest.

## For more information

(i) Advances in Small Trials Design for Regulatory Innovation and Excellence (ASTERIX): http://www.asterix-fp7.eu/
(ii) AFM‐Téléthon: https://www.afm-telethon.fr/
(iii) Australian Genomics Health Alliance: https://www.australiangenomics.org.au/
(iv) Biobanking and Biomolecular Resources Research Infrastructure (BBMRI): http://www.bbmri-eric.eu/
(v) Care4Rare: http://care4rare.ca/
(vi) Centers for Mendelian Genomics: http://mendelian.org/
(vii) E‐Rare: http://www.erare.eu/
(viii) EURenOmics: https://eurenomics.eu/
(ix) European Medicines Agency (EMA): https://www.ema.europa.eu/
(x) European Unified Patient Identity Management (EUPID): https://eupid.eu/
(xi) Find‐A‐Cure: https://www.findacure.org.uk/
(xii) Fondazione Telethon: http://www.telethon.it/en
(xiii) Food and Drug Administration (FDA): https://www.fda.gov/
(xiv) Genomics England: https://www.genomicsengland.co.uk/
(xv) Global Alliance for Genomics and Health (GA4GH): https://www.ga4gh.org/
(xvi) Human Phenotype Ontology (HPO): https://hpo.jax.org/app/
(xvii) Human Variome Project (HVP): http://www.humanvariomeproject.org/
(xviii) Innovation in Small Population Research (InSPiRe): https://warwick.ac.uk/fac/sci/med/research/hscience/stats/currentprojects/inspire/
(xix) Innovative Medicines Initiative (IMI): https://www.imi.europa.eu/
(xx) Integrated Design and Analysis of clinical trials in small sample population groups (IDeAl): https://www.ideal.rwth-aachen.de/
(xxi) International Rare Diseases Research Consortium (IRDiRC): http://www.irdirc.org/
(xxii) IRDiRC Recognized Resources (IRR): http://www.irdirc.org/research/irdirc-recognized-resources/
(xxiii) IRDiRC Task Forces: http://www.irdirc.org/activities/task-forces/
(xxiv) Matchmaker Exchange (MME): https://www.matchmakerexchange.org/
(xxv) National Organization for Rare Disorders (NORD): https://rarediseases.org/
(xxvi) NeurOmics: https://www.neuromics.com/
(xxvii) Online Mendelian Inheritance in Man (OMIM): https://www.omim.org/
(xxviii) Orphacodes: http://www.orphadata.org/
(xxix) Orphanet: https://www.orpha.net/
(xxx) PatientsLikeMe: https://www.patientslikeme.com/
(xxxi) Public Population Project in Genomics and Society (P^3^G): http://p3g2.org/
(xxxii) RareConnect: https://www.rareconnect.org/en
(xxxiii) Rare Diseases Clinical Research Network (RDCRN): https://ncats.nih.gov/rdcrn
(xxxiv) Rare Genomics Institute: https://www.raregenomics.org/
(xxxv) RE(ACT): https://react-community.org/
(xxxvi) Solve‐RD: http://solve-rd.eu/
